# Regulation of Cell Cycle-Related Damage/Repair Mechanism and Oxidative Stress Status by Oroxylin A in Hepatocellular Carcinoma Cells

**DOI:** 10.3390/ijms26188942

**Published:** 2025-09-13

**Authors:** Fatma Seçer Çelik, Safaa Altveş, Canan Eroğlu Güneş

**Affiliations:** 1 Department of Medical Biology and Genetics, Faculty of Medicine, Ankara Medipol University, 06050 Ankara, Turkey; 2 Science and Technology Research and Application Center (BITAM), Necmettin Erbakan University, 42005 Konya, Turkey; safaa.e.t@gmail.com; 3 Department of Medical Biology, Faculty of Medicine, Necmettin Erbakan University, 42005 Konya, Turkey; cananeroglu88@gmail.com

**Keywords:** hepatocellular carcinoma, DNA damage, 8-OHdG, redox hemostasis, Oroxylin A

## Abstract

Hepatocellular carcinoma is a progressive tumor with an aggressive nature. Despite many treatment options, survival rates remain low. In this study, the effect of Orox A on hepatocellular carcinoma cells was investigated. Hep3B cells were treated with a range of Orox A, and cell viability was assessed by MTT assays. Subsequent analyses using RT-qPCR demonstrated alterations in the expression of DNA damage and repair genes. To determine the damage in cancer cells, the amount of 8-OHdG and NO in the cells was measured by the ELISA method. Additionally, total antioxidant/oxidant status was measured, and the OSI value was calculated. Flow cytometry analysis was conducted to ascertain the specific cell cycle phase. The IC50 dose of Orox A for hepatocellular carcinoma cells was calculated to be 1385 μM at 24 h. According to the gene expression analysis results, NEIL1, OGG1, ATM and ATR gene expressions increased significantly, while APEX1 gene expression decreased significantly. The amount of 8-OHdG dramatically increased in cancer cells treated with Orox A, whereas the level of NO significantly decreased. Total antioxidant status (TAS) and total oxidant status (TOS) were significantly decreased in Orox A-applied cancer cells, and the oxidative stress index (OSI) was significantly increased. Flow cytometry analysis revealed that Orox A treatment caused G2/M phase arrest in the cell cycle. These findings collectively suggest that Orox A exerts cytotoxic effects on Hep3B cells through mechanisms involving DNA damage, oxidative stress, and cell cycle modulation, making it a promising candidate for further anticancer therapy development.

## 1. Introduction

Hepatocellular carcinoma (HCC) is characterized by strong tumor heterogeneity and aggressive cell processes, leading to significant tumor invasion, metastasis, poor outcomes, and high mortality [1]. Even though patients with HCC have access to a wide range of therapeutic options, postoperative recurrence and metastasis continue to reduce overall survival. In order to identify new treatment targets and enhance the prognosis for HCC patients, it is crucial to understand the molecular mechanisms underlying the development and progression of HCC.

The O-methylated flavone bioactive component oroxylin A (Orox A; 5,7-dihydroxy-6 methoxyflavone) is extracted from the roots of the Scutellariae plant [2]. A number of biopharmaceutical researchers have shown an interest in Orox A, a type of bioflavonoid in recent years. As a natural product, Orox A has several benefits, such as low toxicity and high selectivity between healthy and cancerous cells [3]. The pharmacological effect of Orox A has been studied both in vitro and in vivo, which suggests that it may be used to treat a number of diseases. The most noteworthy of Orox A’s many pharmacological properties and comparatively minimal toxicity have been shown to be its anticancer benefits. In particular, numerous cancer cell lines, such as those from breast cancer [4], hepatoma [5], non-small cell lung cancer [6], and colon cancer [7], exhibit notable growth inhibition when treated with Orox A. Apoptosis [8], cell cycle arrest [9], and migration/invasion inhibitory effects [10] have all been connected to Orox A’s anticancer qualities. Studies have shown that Orox A suppresses general transcription by inhibiting cyclin-dependent kinase 9 (CDK9), thus slowing down the progression of HCC [11,12]. According to a study conducted on HCC, Orox A significantly suppressed migration in cancer cells. This study showed that Orox A blocks TGF-β1/Smad signaling by increasing NAG-1 expression [13]. Growth-associated disorders, including cancer, can result from a malfunctioning cell cycle regulation system, and many tumors have elevated expression of cell cycle genes and the proteins they encode, which act as proliferation markers [14,15].

This study sought to determine how Orox A affected DNA damage/repair genes and, consequently, the cell cycle, as well as how it inhibited the proliferation of the hepatocellular carcinoma cell line Hep3B cells.

## 2. Results

### 2.1. Orox A Inhibited the Proliferation of Hepatocellular Carcinoma Cells

**Figure 1:**
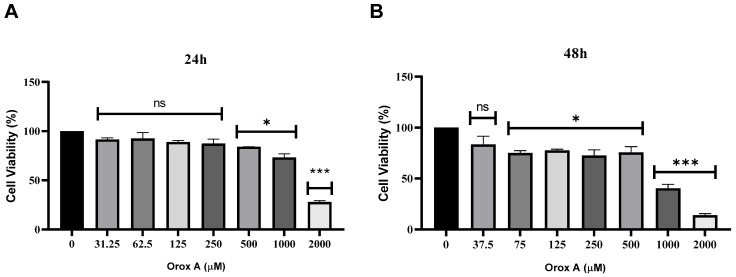
Cytotoxic effect of Orox A on Hep3B cancer cells at (**A**) 24 and (**B**) 48 h. Growth inhibition on Hep3B cells after 24 and 48 h treatment by different concentrations of Orox A, ranging from 0 to 2000 μM, was evaluated via MTT assay. IC_50_ value of Orox A for Hep3B cells was calculated as 1385 μM at 24 h via CompuSyn version 1.0 software. Obtained data was analyzed using ordinary one-way ANOVA test. *p*-values < 0.05 were considered as statistically significant (*). *p*-values less than 0.001 were designated with (***). *p*-values > 0.05 were reported as statistically not significant (ns). Each group had three biological replicates.

Hep3B cells were treated with a concentration range of Orox A (0–2000 μM) for 24 and 48 h. It was found that as the dose increased, the viability of hepatocellular carcinoma cells decreased (Figure 1). Using CompuSyn version 1.0 software, the half maximum inhibitory concentration (IC_50_) of Orox A for Hep3B cells was determined as 1385 μM at 24 h. Thus, Hep3B cells were treated with a concentration of Orox A corresponding to its calculated IC_50_ value in the following experiments.

### 2.2. Orox A Altered the Expression of DNA Damage and Repair Genes in Hepatocellular Carcinoma Cells

**Figure 2:**
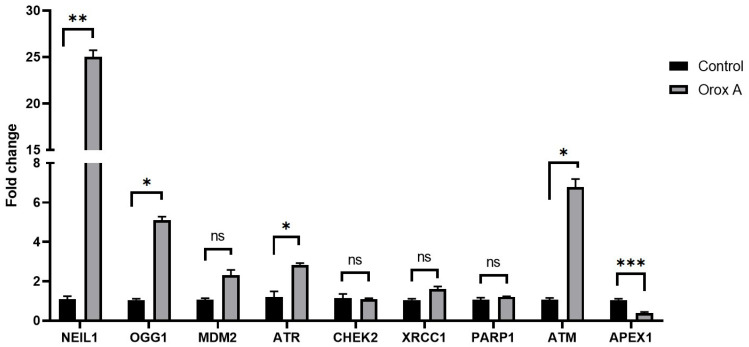
Expression changes of DNA damage and repair pathway-related genes. Effect of Orox A on NEIL1, OGG1, MDM2, ATR, CHEK2, XRCC1, PARP1, ATM, and APEX1 mRNA levels in Hep3B cells after treatment with 1385 μM for 24 h. The relative quantification of the target genes was performed using the 2^(−ΔΔCt)^ method. ACTB was used as a housekeeping gene in RT-qPCR experiments. Bar graphs representing the fold changes in control and treatment groups were drawn with GraphPad Prism (Version 8.0.2, GraphPad software Inc., San Diego, CA, USA). Obtained data was analyzed using Student’s *t*-test. *p*-values < 0.05 were considered as statistically significant (*). *p*-values less than 0.01 were designated with (**) and less than 0.001 were designated with (***). *p*-values > 0.05 were reported as statistically not significant (ns). Each group had three biological replicates.

Based on our RT-qPCR results of Orox A treated Hep3B cells, statistically significant changes were obtained in the expression levels of NEIL1 (nei like DNA glycosylase 1), OGG1 (8-oxoguanine DNA glycosylase), ATR (ATR checkpoint kinase), ATM (ATM serine/threonine kinase) and APEX1(apurinic/apyrimidinic endodeoxyribonuclease 1) genes, especially those involved in the DNA damage pathways, as compared to the control cells (Figure 2, *p* < 0.05). Therefore, Orox A treatment has been shown to trigger DNA damage in hepatocellular carcinoma cells.

As a result of gene expression analysis, mRNA levels of ATM and ATR increased significantly (*p* < 0.05) which are related to the mechanism of the DNA double strand breaks, the change in the expression levels of PARP1 (poly(ADP-ribose) polymerase 1) and XRCC1 (X-ray repair cross complementing 1) genes were not significant which are related to the DNA single strand breaks (Figure 2, *p* > 0.05). On the other hand, a remarkable decrease in the APEX1 gene was detected (Figure 2, *p* < 0.001) in Orox A-treated cells as compared to the control group. Moreover, the levels of OGG1 and NEIL1, giving a hint about the oxidative stress, were found to significantly increase in Orox A-treated cells (Figure 2, *p* < 0.05). Our results clearly indicated that the treatment of Hep3B cells with Orox A induces DNA damage and oxidative stress.

### 2.3. Orox A Application Increases the Amount of DNA Damage Product 8-OHdG in Hepatocellular Carcinoma Cells

**Figure 3:**
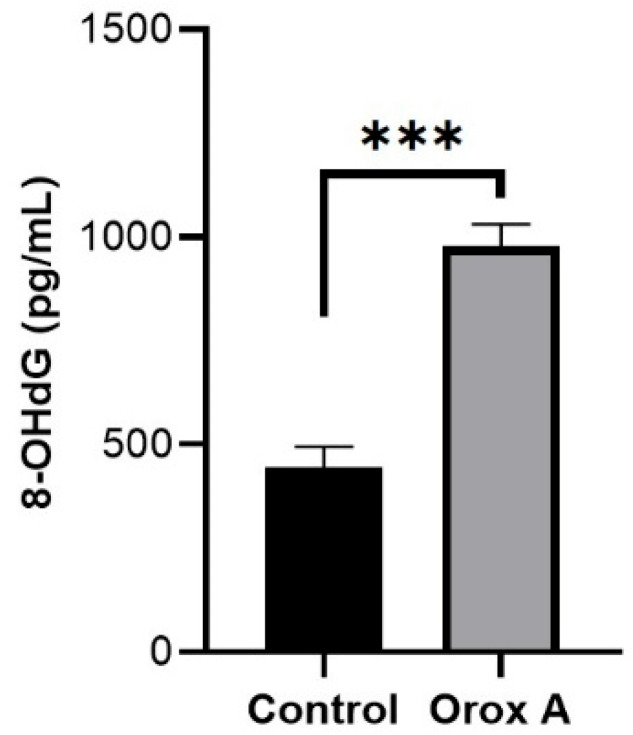
The amount of 8-OHdG in 1385 μM Orox A-treated and control cells in hepatocellular carcinoma cells. The graph was depicted using GraphPad Prism (Version 8.0.2, GraphPad software Inc., San Diego, CA, USA). The results were indicated as *p*-values < 0.001 were designated with (***).

The amount of 8-OHdG increased significantly in hepatocellular carcinoma cells treated with Orox A (Figure 3; Table 1, *p* < 0.001).

### 2.4. Orox A Suppressed Nitric Oxide Synthesis in Hepatocellular Carcinoma Cells

**Figure 4:**
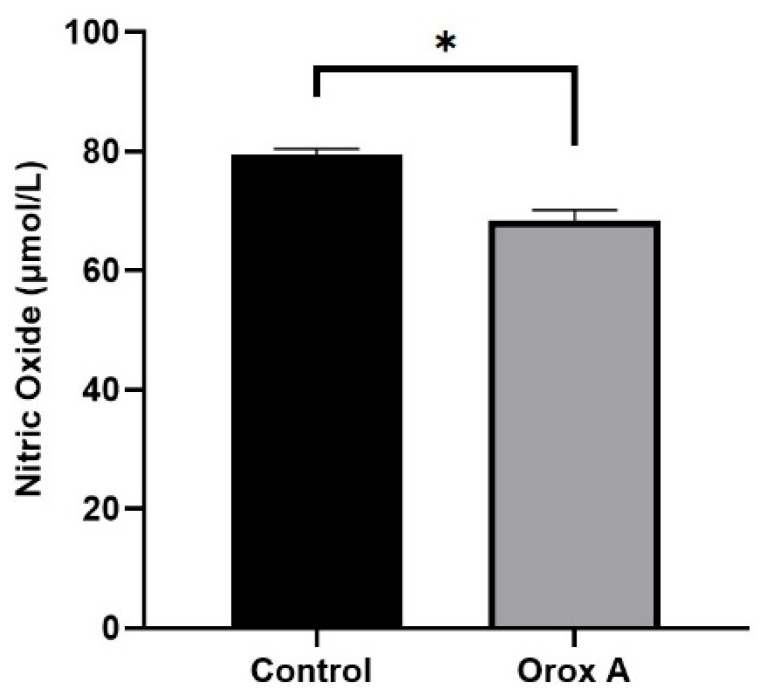
The amount of NO in 1385 μM Orox A-treated and control cells in hepatocellular carcinoma cells. The graph was depicted using GraphPad Prism (Version 8.0.2, GraphPad software Inc., San Diego, CA, USA). The results were indicated as *p*-values < 0.05 were designated with (*).

Our ELISA results indicated that nitric oxide was significantly inhibited by Orox A treatment in Hep3B cells (Figure 4; Table 2, *p* < 0.05).

### 2.5. The Effect of Orox A Treatment on Redox Balance of Hepatocellular Carcinoma Cells

**Figure 5:**
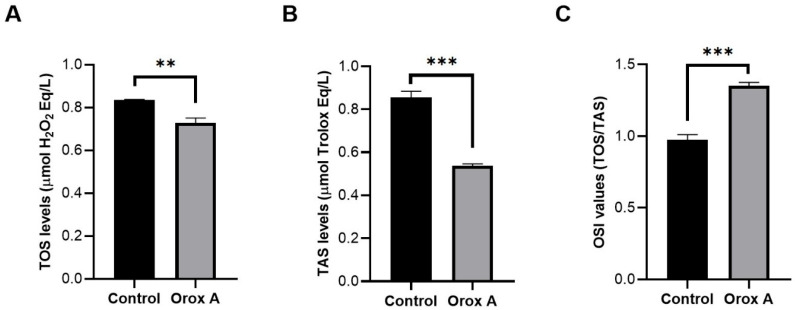
Amounts of TAS and TOS in Hep3B cells treated with 1385 μM Orox A (**A**,**B**), and ratio of TAS and TOS to each other (**C**). The levels of TAS (**A**) and TOS (**B**), as well as the calculated TOS/TAS ratio, OSI values (**C**), in the control and Orox A-treated Hep3B, hepatocellular carcinoma cell line, were depicted using GraphPad Prism (Version 8.0.2, GraphPad software Inc., San Diego, CA, USA). The results were indicated as *p*-values between 0.01 and 0.001 were designated with (**), <0.001 were designated with (***).

Orox A-treated Hep3B cells had significantly low TOS levels, which significantly decreased in Odo A-treated hepatocellular carcinoma cells as compared to control cells, indicating elevated oxidative stress upon our treatment (Figure 5A, *p* < 0.05). TOS levels represent pro-oxidant status in cells. Based on our data, the fact that TAS levels were lower in the experimental group than in control cells indicates that the antioxidant capacity was reduced. (Figure 5B, *p* < 0.05). Moreover, the calculated OSI values were also significantly higher in the treatment group than in the control samples (Figure 5C; Table 3, *p* < 0.05). Low TAS level, low TOS level, and high OSI value indicated an overall oxidative stress in Orox A-treated hepatocellular carcinoma cells (Table 3).

### 2.6. Orox A Induces Cell Cycle Arrest at the G2/M Phase

**Figure 6:**
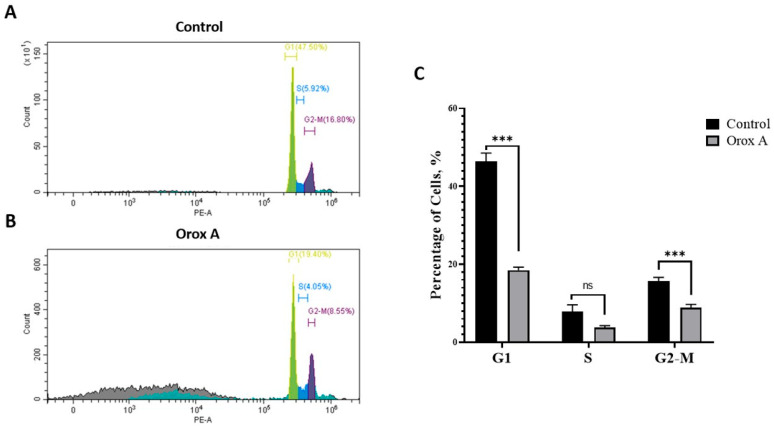
Effect of Orox A on cell cycle distribution in Hep3B. Cells were untreated (Control, (**A**)) or treated with 1385 μM Orox A (**B**), and bar graphics were analyzed by flow cytometry after DNA staining with PI (**C**). The percentage of cells in G1, S, and G2-M phases was determined from DNA content histograms. Data represent mean ± SD from three independent experiments. Statistical analysis was performed using multiple unpaired *t*-tests (one per cell cycle phase), with Welch’s correction for unequal variances and Bonferroni-Dunn correction for multiple comparisons. The results were indicated as *p*-value 0.001 was designated with (***). *p*-values > 0.05 were reported as statistically not significant (ns).

Cell cycle distribution was assessed by flow cytometry using the PI method. As shown in Figure 6, treatment with Orox A significantly altered the distribution of cells across the cell cycle phases compared to the control group. In the control group, a higher percentage of cells were observed in the G1 phase (approximately 47%) compared to the Orox A group (approximately 18%), indicating a significant reduction in G1-phase cells upon treatment. In contrast, the proportion of cells in the S phase showed a slight increase, but the difference was not statistically significant. Importantly, a marked accumulation of cells was observed in the G2/M phase in the Orox A group (around 9%) compared to the control group (approximately 16%), which also showed a statistically significant difference. These results suggest that the Orox A induces cell cycle arrest primarily at the G2/M phase.

## 3. Discussion

Nowadays, many treatment options are used against cancer, which is increasingly common. Overall survival, especially in aggressive cancers, is decreasing despite these treatment options. For this reason, it is vital to discover new agents that are less toxic to healthy cells but highly toxic to cancer cells. Anti-cancer drug studies are on phytochemical compounds obtained from many herbal extracts. The effect of phytochemicals on cancer cells may occur through additional cellular mechanisms. So, only viability analysis is insufficient to reveal the mechanism of action of the agent used. Orox A is extracted from the roots of the Scutellariae plant. In this study, we investigated the effect of Orox A on DNA damage and repair mechanism genes in hepatocellular carcinoma cells, how it affects the oxidative status in the cell, and what changes it creates in the cell cycle.

Orox A has been demonstrated in a prior work to induce apoptosis in cancer cells through the mitochondrial route in HepG2 cells. Reactive oxygen species produced by Orox A in HepG2 cells were found to activate mitochondrial permeability transition pores (MPTP; [16]). In another study, the inhibitory effect of Orox A on viability was demonstrated in three different colorectal cancer cell lines [17]. Consistent with the literature, the current study showed that Orox A is also toxic in Hep3B cells (Figure 1). Orox A has been shown to increase H_2_O_2_ levels in HepG2 cells compared to normal cells. This result was associated with the formation of excessive H_2_O_2_, stimulating the UPR pathway and suppressing the Akt pathway [18]. Thus, it can be concluded that Orox A selectively induces cytotoxicity in cancer cells, not normal cells. In addition, it has been shown that Orox A exhibits a cytotoxic effect at 50 µM in synergistic effects with doxorubicin [12], but is toxic in HepG2 cells at approximately 200 µM for 36 h [18]. In our study, a higher concentration (1385 µM) was used for a shorter period.

In this study, expression analysis of DNA damage and repair genes was performed to examine the effect of Orox A on cell DNA. According to the results, it was determined that the expressions of NEIL1, OGG1, ATR, ATM, and APEX1 genes changed significantly (Figure 2; *p* < 0.05). When the gene expression changes were examined one by one, it was seen that NEIL1 and OGG1 genes, which are effective in DNA single-strand breaks (SSBs), increased significantly with Orox A treatment. Numerous endogenous or exogenous causes, such as mistakes in DNA replication, the activity of enzymes like DNA glycosylase and topoisomerase, ROS, ultraviolet (UV) and ionizing radiation (IR), and chemicals, continuously damage human genomic DNA [19,20]. Several dozen oxidized DNA bases, base mismatches, DNA interstrand crosslinks, and strand breaks, including single-strand breaks and double-strand breaks (DSBs), are among the different forms of DNA damage that are caused by different sources. The unrepaired DSB may be enough to cause cell death, making DSBs the most deadly type of damage [21]. In our study, the significant increase in ATM and ATR gene expressions in the Orox A group is associated with the formation of DSBs and the stimulation of ATM/ATR genes. ATM protein-induced CHEK2 expression showed an insignificant decrease (Figure 2; *p* > 0.05). Expression changes of PARP1 and XRCC1 were found to be insignificant in relation to SSBs. However, in the PARP1/APEX1/XRCC1 axis, only the expression change of APEX1 showed a significant decrease. In this case, when the repair mechanism induced by Orox A is examined, it can be said that it causes DSBs rather than SSBs. As is known, chemotherapeutics generally cause DSBs in DNA [22].

By stimulating tumor-cell invasion, proliferation, and the production of angiogenic factors, NO generated from tumor cells accelerates the growth of tumors. Despite contradictory results in the literature, the inducible isoform of NOS (iNOS), which generates high NO concentrations, drives neoplastic transformation in oncogene- and chemical-induced carcinogenesis models [23,24]. According to the results obtained in our study, the amount of NO decreased significantly in the group that applied Orox A (Figure 4). NO, a factor that triggers both vascularization and invasion of cancer cells, was reduced in the cells by Orox A. This suggests that Orox A may have an anti-invasive effect on cancer cells.

As a measure of oxidative DNA damage, 8-oxo-7, 8-dihydroguanine (8-oxoG), a guanine C-8 hydroxylation mediated by OH, is the oxidative DNA damage most often seen. Interestingly, mitochondrial DNA has a higher 8-oxoG lesion than nuclear DNA, indicating that mitochondrial DNA is more vulnerable to oxidative damage [25]. In this study, we found that the amount of 8-OHdG increased significantly in cancer cells treated with Orox A (Figure 3). At the same time, a high OSI value indicated an overall oxidative stress in Orox A-treated hepatocellular carcinoma cells (Figure 5C). While an acceptable amount of free radicals maintains the essential processes of cancer cell survival, a high level of ROS promotes cell death by inducing apoptosis and/or autophagy [26,27]. When OGG1 detects oxidative DNA damage, it excises 8-oxoG, providing a substrate for apurinic endonuclease 1 (APE1) and starting the repair process [28]. According to our results, the increase in OGG1 gene expression correlates with the significant increase in 8-OHdG.

Many anticancer chemicals, including doxorubicin, 5-fluorouracil, and cisplatin [29], were developed as a result of the idea that anticancer treatments should target DNA. In this study, we evaluated the effects of Orox A on cell cycle progression using flow cytometry. The results clearly demonstrated that Orox A significantly altered the cell cycle profile, characterized by a marked reduction in the G1 population and a significant accumulation of cells in the G2/M phase (Figure 6). This suggests that Orox A induces G2/M phase arrest, a common hallmark of compounds that interfere with cell cycle regulatory mechanisms or induce DNA damage.

Cell-cycle checkpoint proteins identify high amounts of DNA damage and, when activated, cause cell-cycle arrest, which stops damaged DNA from being transferred during mitosis. Replication-associated DNA DSBs, one of the most harmful types of DNA lesions, can result from DNA lesions that happen during the S phase of the cell cycle. They also prevent replication fork progression. Cell death could occur if the damaged DNA cannot be adequately repaired. In addition to having a relaxed ability to sense and repair DNA damage, cancer cells are more likely to be able to ignore cell-cycle checkpoints, which enables them to proliferate at high rates. This makes them more vulnerable to DNA damage because replicating damaged DNA increases the risk of cell death [30]. G2/M arrest is often associated with cellular responses to genotoxic stress or microtubule disruption. It serves as a protective mechanism to prevent cells with damaged or unreplicated DNA from entering mitosis [31]. Several anticancer agents, including natural products, exert their antiproliferative effects by inducing G2/M arrest [32]. Similar to Orox A, flavonoid derivatives and polyphenolic compounds have been shown to induce G2/M phase arrest by modulating cyclin-dependent kinases (CDKs) and activating checkpoint kinases such as Chk1 and Chk2 [33,34]. Taken together, our findings suggest that Orox A disrupts cell cycle progression and induces a cytostatic effect via G2/M arrest. This supports its potential development as a candidate for anticancer therapy.

## 4. Materials and Methods

### 4.1. Cell Culture and Chemicals

Hep3B (ATCC^®^ HB-8064™) hepatocellular carcinoma cell line was obtained from ATCC (Manassas, VA, USA). These cells were grown in EMEM (Eagle’s Minimum Essential Medium) medium with 10% FBS, 1% penicillin/streptomycin, and 2 mM L-glutamine at 37 °C in a cell culture incubator under a proliferating 5% CO_2_ environment. The cells were used in the subsequent studies after confluence reached 80%.

Orox A (Cat#82615) has been commercially obtained from PhytoLab, Vestenbergsgreuth, Germany. Orox A dissolving process has been carried out using dimethylsulfoxid (DMSO). First, 1.14 g of Orox A was completely dissolved in 1 mL of DMSO in a controlled manner until no single particle was visible in a solution. Then, 10 mL of culture medium was added to DMSO-dissolved Orox-A solution, and initial stock solution was prepared.

### 4.2. MTT Assay

A 96-well microplate was inoculated with 100 μL/well after the cell suspension concentration was adjusted to 2.5 × 10^4^ cells/mL. The cells were kept in the incubator for twenty-four hours. The cells were treated with 8 different doses of Orox A (0–2000 μM), the cells were cultivated for 24 and 48 h in the incubator. Then, for four hours, 100 μL of 0.5 mg/mL MTT solution was applied to each well. MTT solution was withdrawn from the wells. Moreover, 100 μL of DMSO is added to each well and shaken for 10 min. The microplate reader picked up the OD value at 570 nm, which is the detection wavelength.

### 4.3. RNA Isolation and cDNA Synthesis

Total RNA isolation was performed from Hep3B cells for mRNA-level expression studies. For this purpose, 1 mL of RiboEx total RNA isolation solution (GeneAll, Hwaseong-si, Republic of Korea) was added to the cells in 6-well plates. Homogenate was transferred in Eppendorf tubes. After incubation for 10 min at room temperature, 200 μL of chloroform was added to each Eppendorf tube and pipetted again, then incubated for 15 min at room temperature. Then, it was centrifuged at 15,000× *g* for 20 min at +4 °C, and the supernatant was collected and taken into separate Eppendorf tubes. Moreover, 250 μL of isopropanol and 250 μL NaCl (1.2 M) were added and kept at room temperature for 10 min. After centrifuging at 15,000× *g* for 30 min at +4 °C, the supernatant was carefully discarded, and the pellet was washed using 70% ethanol. After centrifugation for 10 min at +4 °C, the pellet was dried briefly and dissolved with 30 μL nuclease-free water [35]. The quality and quantity of total RNAs isolated from the control and dose groups were measured by using Nanodrop at 260/280 nm UV. cDNAs were synthesized from total RNAs using the protocol of the manufacturer (Bio-Rad iScript cDNA Synthesis, Hercules, CA, USA).

### 4.4. RT-qPCR Analysis

SyberGreen-I mix was completed with 1X in qPCR mixes, 5 pmol forward and 5 pmol reverse primers (Table 4), 2 μL cDNA as template, and sterile ddH_2_O with a total volume of 20 μL. The mix was placed onto an RT-qPCR platform (Bio-Rad CFX Connect Real-Time PCR System), the heat profile of the reaction was set to +95 °C for 10 min, 40 cycles (95 °C 30 s, 60 °C 30 s, 72 °C 30 s). Later, melting curve analysis was performed by heating it at 95 °C for 1 min and decreasing the temperature to 55 °C gradually until 95 °C and by making optical measurements at every 0.5 °C increase. Data from optical measurements in the RT-qPCR reaction were recorded, and Ct values were normalized using ACTB as a reference gene.

### 4.5. Measurement of the DNA Damage Product 8-OHdG

Human 8-OHdG kit (Bostonchem, Cat# BLS-8533Hu, Cambridge, MA, USA) was used to detect DNA damage. Cell lysates were prepared as recommended by the manufacturer. Moreover, 50 μL samples were placed in 96-well pre-coated microplate. Additionally, 50 μL biotinylated-conjugate solution was added to each well. It was incubated at 37 °C for 1 h. It was washed 3 times with wash buffer. Then, 100 μL streptavidin-HRP solution was added to each well. It was incubated again at 37 °C for 1 h. Wells were washed 3 times with wash buffer again. Moreover, 90 μL TMB substrate solution was added to each well and covered with a plate cover and incubated for 20 min at 37 °C. Furthermore, 50 μL stop reagent was added, and OD measurement was performed at 450 nm.

### 4.6. Nitric Oxide (NO) Measurement Using ELISA

The General Nitric Oxide Assay Kit (Cat#MBS8243214, MyBioSource, San Diego, CA, USA) was used to test the levels of nitric oxide (NO). After removing Hep3B cells from culture plates, the supernatant was discarded after centrifugation. Following the addition of 500 μL of distilled water, the mix was homogenized. It was centrifuged for 20 min at 4 °C at 12,000× *g*. After the addition of 250 μL of assay buffers 1 and 2, the mixture was centrifuged at 10,000× *g* for 10 min at 4 °C. A clean tube was filled with the supernatant. Each well of a 96-well plate contained 50 μL of the sample. Ten minutes of incubation was performed following the addition of 50 μL of dye reagent A. Finally, 50 μL of Dye Reagent B was added, and the mixture was incubated for five minutes. At 550 nm OD, the absorbance was measured and recorded.

### 4.7. Biochemical Evaluation of Redox Equilibrium

Human TAS (SanRedBio Technology Campany, Cat#201-12-7412, Shanghai, China) and TOS (SanRedBio Technology Campany, Cat# 201-12-5539, China) ELISA kits were used to measure the total antioxidant status (TAS) and total oxidant status (TOS) of Orox A-treated and control Hep3B cells. By dividing TOS by TAS levels, the oxidative stress index (OSI) value was determined. The automated colorimetric method created by Erel (2004) [36] was used to measure the TAS levels in the control and experimental groups in accordance with the manufacturer’s instructions. Through the formation of hydroxyl free radicals (OH·), the examined sample’s antioxidative capacity is measured against possible reactive oxygen species (ROS) reactions. The results obtained were reported as a quantity in µmol Trolox Eq/L [36]. The automated colorimetric methods created by Erel (2004) were also used to ascertain the TOS levels in the control and experimental groups. The quantity of total oxidant molecules present in the sample was connected with the color intensity as determined by spectrophotometry. The assay was calibrated using hydrogen peroxide (H_2_O_2_) free radicals, and the results were represented as a quantity in µmol H_2_O_2_ Eq/L [36]. The OSI value was computed as follows: the OSI value was expressed in arbitrary units as a measure of quantity and was determined as the ratio of TOS to TAS levels [37].

### 4.8. Determination of Cell Cycle Stage by Flow Cytometry

Hep3B cells were seeded on 6-well plates 5 × 10^5^ cells per well. Cells were incubated and treated as described above. To evaluate cell cycle, cells were collected and fixed with 70% ethanol, then treated with 10 µL PureLink™ RNase A (Invitrogen, Cat# 12091039, Waltham, MA, USA) for 20 min at 37 °C, and finally, 200 µL of propidium iodide (Thermo Scientific, Cat# 440300250, Waltham, MA, USA). Cells were analyzed through Beckman Coulter^®^ Flow cytometry device and CytExpert^®^ software (version 2.5.0.77).

### 4.9. Statistical Analysis

The 2^(−ΔΔCt)^ technique was used to calculate relative fold changes in gene expression levels of samples for the RT-qPCR study. The statistical analysis of RT-qPCR results, ELISA data, TAS and TOS levels, and OSI values was performed via the GraphPad Prism statistics tool (Version 8.0.2; GraphPad software Inc., San Diego, CA, USA). The parametric variables were analyzed through Student’s *t*-test, while nonparametric variables were compared using the Mann–Whitney U test. In every analysis, *p*-values < 0.05 were considered as statistically significant.

## 5. Conclusions

The IC_50_ of Orox A for hepatocellular carcinoma cells was determined to be 1385 μM in 24 h, which was used in our experiments to test the hypothetical question of the current study. Based on RT-qPCR analysis of Orox A-treated hepatocellular carcinoma cells, significant increases in the mRNA transcript levels of NEIL1, OGG1, ATR, and ATM were observed. However, the APEX1 gene expression level was significant. Therefore, Orox A treatment of hepatocellular carcinoma cells induced 8-OHdG products. Moreover, we found that the amount of intracellular NO decreased significantly. Furthermore, the increase in OSI value in Orox A-applied cells suggests that increased oxidative stress indirectly leads to cancer cell death. Finally, Orox A has been shown to have a therapeutic effect on hepatocellular carcinoma cells in vitro. Further in vivo studies will be conducted to more clearly demonstrate the physiological effects of Orox A in cancer treatment.

## Figures and Tables

**Table 1:** The amounts of 8-OHdG in Orox A-treated and control in hepatocellular carcinoma cells.

	Control Group	Orox A-Treated Group	*p*-Values
**8-OHdG** (pg/mL)	446 ± 27.75	979 ± 29.57	<0.001 (***)

The results were indicated as ± SEM, and *p*-values < 0.001 were designated with (***).

**Table 2:** The amount of NO in Orox A-treated hepatocellular carcinoma cells and in control.

	Control Group	Orox A-Treated Group	*p*-Values
**NO** (μmol/L)	79.37 ± 0.6117	68.32 ± 1.0666	<0.05 (*)

The results were indicated as ±SEM, and *p*-values < 0.05 were designated with (*).

**Table 3:** Oxidant and antioxidant parameters in Orox A-treated hepatocellular carcinoma cells and in control were provided. TAS, total antioxidant status; TOS, total oxidant status; OSI, oxidative stress index.

	Control Group	Orox A-Treated Group	*p*-Values
**TAS** (μmol Trolox Eq/L)	0.8567 ± 0.016	0.5380 ± 0.005	<0.001 (***)
**TOS** (μmol H_2_O_2_ Eq/L)	0.8350 ± 0.017	0.7280 ± 0.013	0.001 (**)
**OSI** (arbitrary unit)	0.9750 ± 0.021	1.353 ± 0.013	<0.001 (***)

The results were indicated as ±SEM, and *p*-values between 0.01 and 0.001 were designated with (**); <0.001 was designated with (***).

**Table 4:** Primers used in this study.

Gene	Forward Primer (5′-> 3′)	Reverse Primer (5′-> 3′)	PCR (bp)
***NEIL1***	TCCTGTACCGGCTGAAGAT	TTCTGCAGCTTGGTCCTTATC	121
*OGG1* *	GGCTCAACTGTATCACCACTGG	GGCGATGTTGTTGTTGGAGGAAC	143
***MDM2***	CCTACTGATGGTGCTGTAACC	TGTGCACCAACAGACTTTAATAAC	107
*ATR* *	GGAGATTTCCTGAGCATGTTCGG	GGCTTCTTTACTCCAGACCAATC	100
***CHEK2***	GCGCCTGAAGTTCTTGTTTC	GGATACCCACTAAGGCAGATAAA	101
*XRCC1* *	CGGATGAGAACACGGACAGTGA	GAAGGCTGTGACGTATCGGATG	152
*PARP1* *	CCAAGCCAGTTCAGGACCTCAT	GGATCTGCCTTTTGCTCAGCTTC	123
***ATM***	GCCGTCAACTAGAACATGATAGA	CCTTGTTTGGAATCTGAATGCC	121
*APEX1* *	CTGCTCTTGGAATGTGGATGGG	TCCAGGCAGCTCCTGAAGTTCA	148
***ACTB***	TGGCTGGGGTGTTGAAGGTCT	AGCACGGCATCGTCACCAACT	179

* Designated by https://www.origene.com/, accessed on 22 August 2025.

## Data Availability

The data used/analyzed in this current study are available from the corresponding author upon request. Correspondence and requests for materials and data should be addressed to F.S.C.
